# Supplementary material for: C3 complement inhibition prevents antibody-mediated rejection and prolongs renal allograft survival in sensitized non-human primates
Source: Nat Commun. 2021 Sep 15;12:5456. doi: 10.1038/s41467-021-25745-7 (PMC8443599; doi:10.1038/s41467-021-25745-7)
Supplement: Supplementary file 1 — Supplementary Information [file 41467_2021_25745_MOESM1_ESM.pdf]

Supplementary Information

C3 Complement Inhibition Prevents Antibody-mediated Rejection and Prolongs Renal Allograft Survival in Sensitized Non-human Primates

Robin Schmitz[1], Zachary W. Fitch[1], Paul M. Schroder[1], Ashley Y Choi[1], Miriam Manook[1], Janghoon Yoon[1], Ming Song[1], John S. Yi[2], Sanjay Khandelwal[3], Gowthami M. Arepally[3], Alton B. Farris[4], Edimara S. Reis[5], John D. Lambris[5], Jean Kwun[1,*], and Stuart J. Knechtle[1,*]

1 Duke Transplant Center, Department of Surgery, Duke University School of Medicine, Durham, NC
2 Division of Surgical Sciences, Department of Surgery, Duke University, Durham, NC
3 Department of Medicine, Duke University School of Medicine, Durham, NC
4 Department of Pathology, Emory University School of Medicine, Atlanta, GA
5 Department of Pathology and Laboratory Medicine, University of Pennsylvania, Philadelphia, PA

[*] Corresponding authors: Jean Kwun and Stuart J. Knechtle
Email: jean.kwun@duke.edu and stuart.knechtle@duke.edu

Supplementary Figure 1

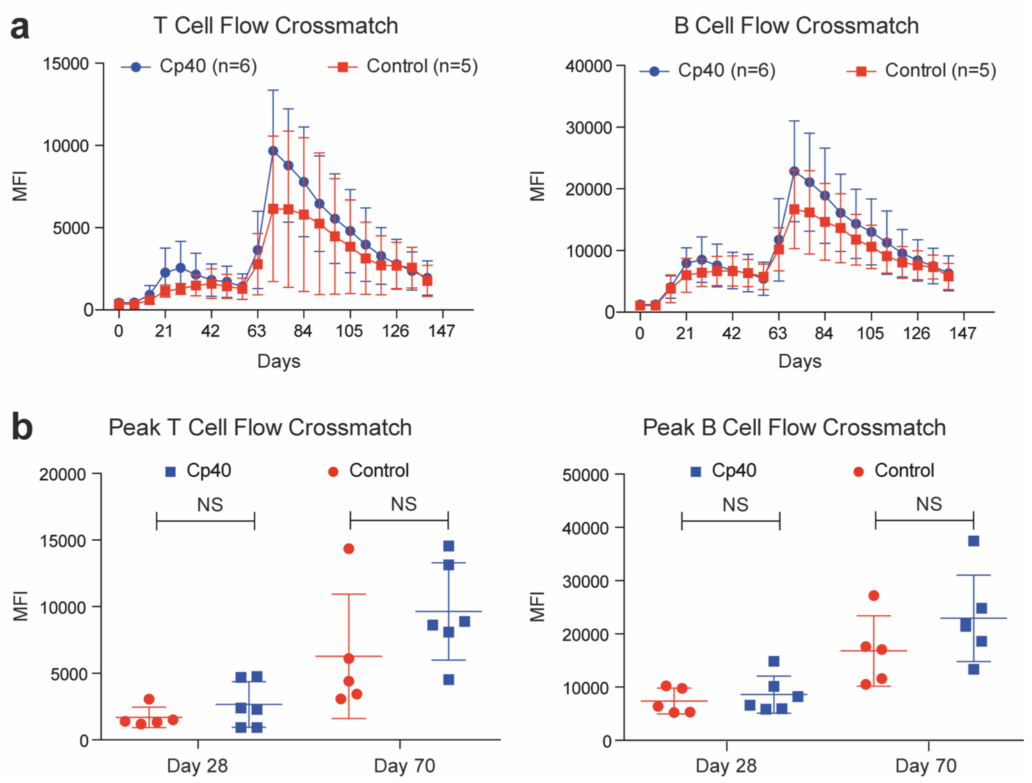

Supplementary Fig. 1: T and B cell flow crossmatch during skin allograft sensitization. **(a)** T and B cell flow crossmatch for control group (red, n=5) and Cp40 group (blue, n=6) throughout the skin allograft sensitization period. Skin transplants were performed 8 weeks apart. **(b)** Peak MFI of T and B cell flow crossmatch after first and second skin transplant for the control (n=5) and Cp40 group (n=6). All data are presented as mean ± SD; N number is reflecting biologically independent animals; NS indicates no statistical significance using two-tailed parametric unpaired t test.

Supplementary Figure 2

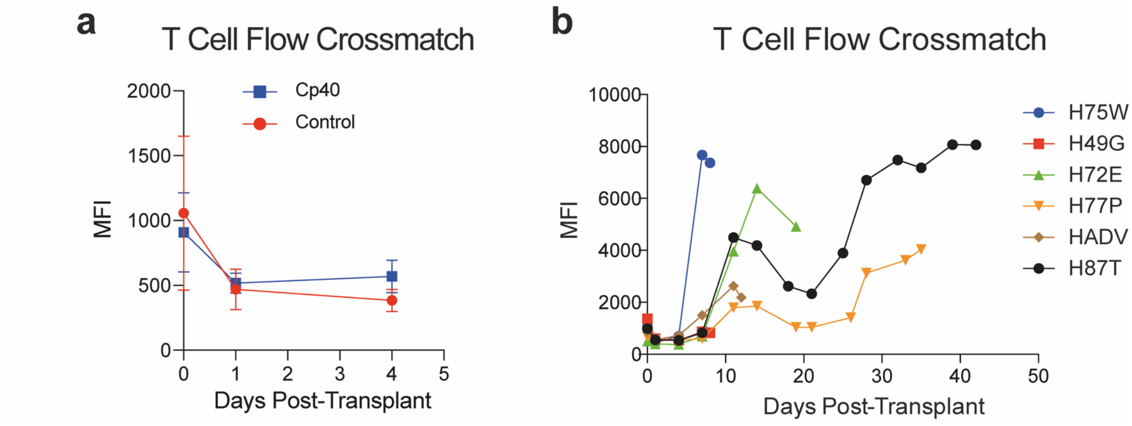

Supplementary Fig. 2: Post-transplant DSA levels. (a) T cell flow crossmatch of the early post-transplant phase (day 0-4) of the control (n=5) and Cp40-treated (n=6) group. Data are presented as mean ± SD. **(b)** T cell flow crossmatch of all Cp40-treated primates (n=6) throughout the study period. N number is reflecting biologically independent animals.

Supplemental Figure 3

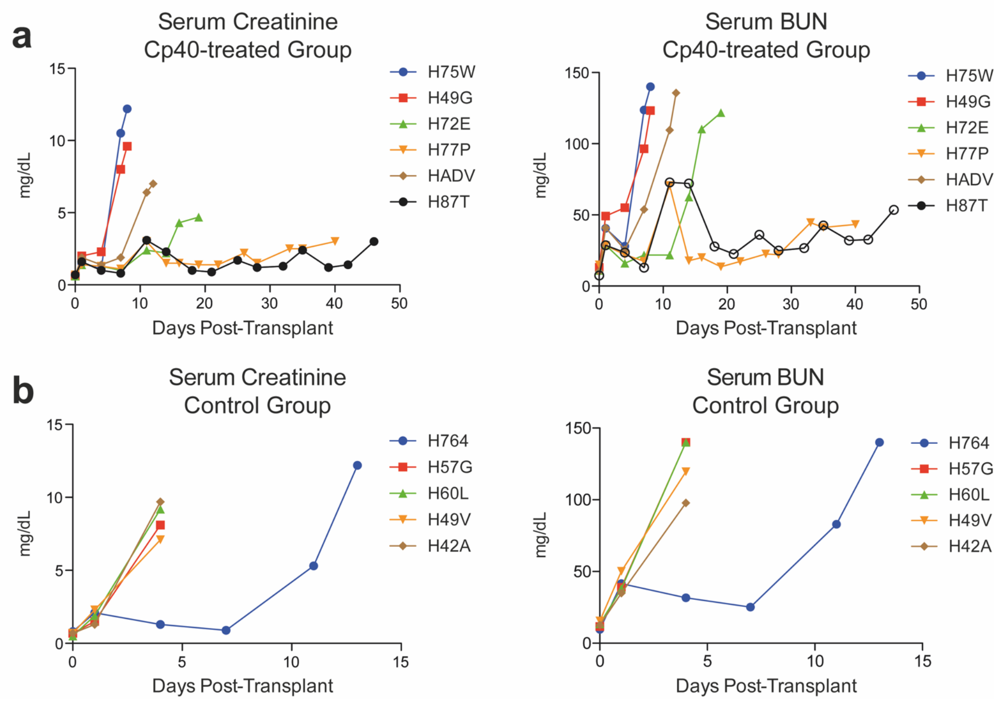

Supplementary Fig. 3: Post-transplant kidney allograft function. (a) Serum creatinine and blood urea nitrogen (BUN) levels for each Cp40-treated primate (n=6) throughout the study period. **(b)** Serum creatinine and blood urea nitrogen (BUN) levels for each control group primate (n=5) throughout the study period. N number is reflecting biologically independent animals.

Supplementary Figure 4

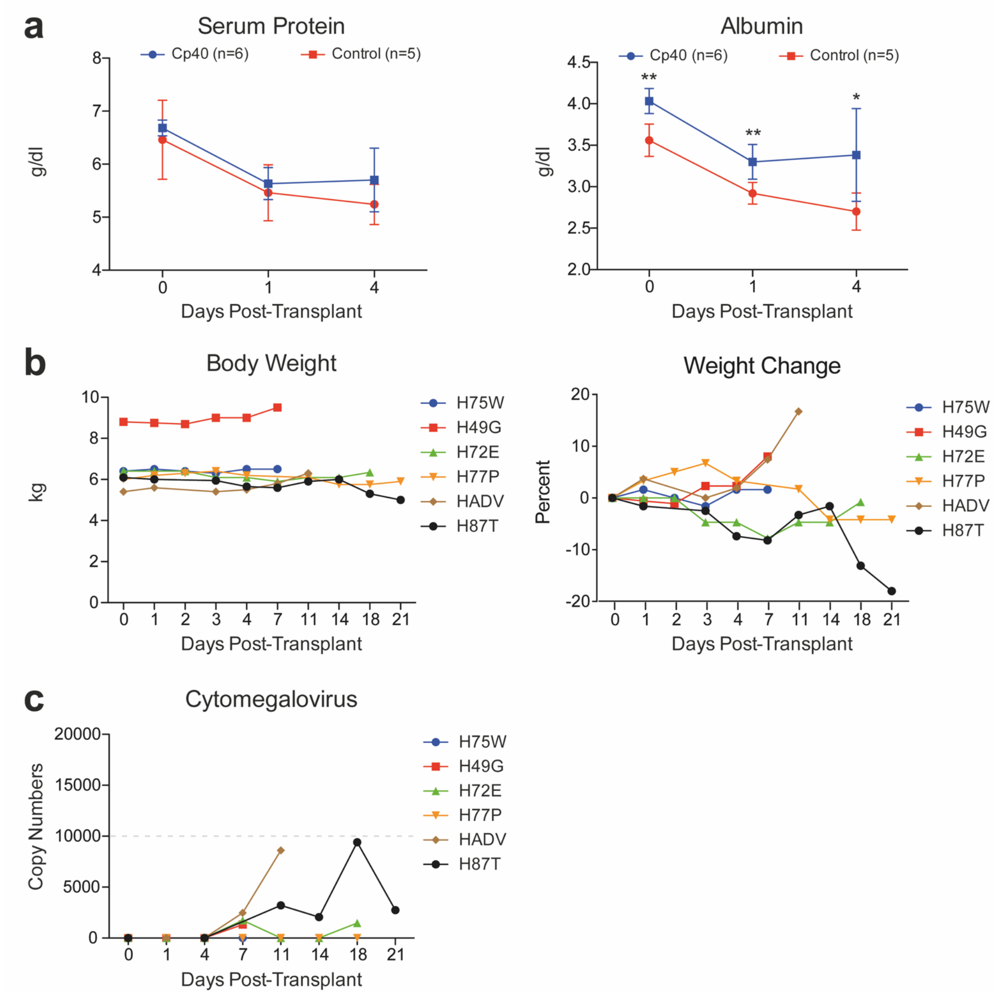

Supplementary Fig. 4: Early post-transplant nutritional status and CMV titer. (a) Post kidney transplant serum protein and albumin levels of the control group (red, n=5) and Cp40-treated group (blue, n=6). Serum albumin levels were significantly higher in Cp40-treated group at post-transplant day 0 (p=0.0014), 1 (p=0.0066), and 4 (p=0.0314) compared to control animals. Data are presented as mean ± SD. **(b)** Post-transplant absolute body weights and relative weight changes of each primate in the Cp40-treated group throughout the study period. **(c)** Post-transplant rhesus CMV titers of each primate in the Cp40-treated group throughout the study period. * indicates p<0.05; ** indicates p<0.01 using two-tailed parametric unpaired t test

Supplementary Figure 5

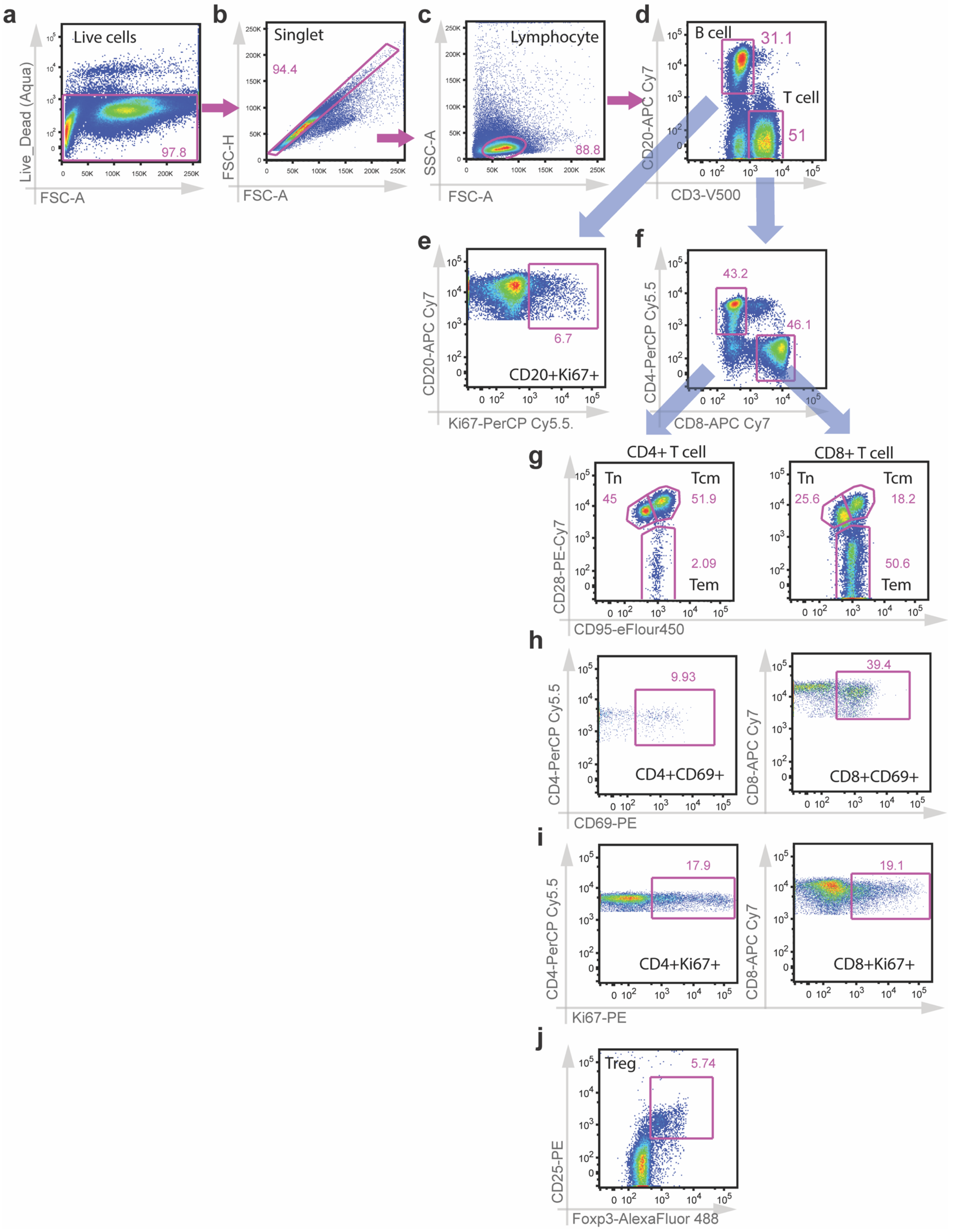

Supplementary Fig. 5: Flow cytometry gating strategy. Representative flow cytometry plots of peripheral blood mononuclear cells showing our gating strategy for **(a)** Live cells. **(b)** Singlet. **(c)** lymphocytes. **(d)** T ($CD3^+$) and B ($CD20^+$) cells. **(e)** Proliferating B ($CD20^+Ki67^+$) cells. **(f)** CD4 ($CD3^+CD4^+$) and CD8 ($CD3^+CD8^+$) T cells. **(g)** Naive ($CD28^+$ $CD95^-$), central

memory ($CD28^+$ $CD95^+$), and effector memory ($CD28^-$ $CD95^+$) cells in CD4 and CD8 T cell populations. **(h)** Recently activated CD4 ($CD69^+CD4^+$) and CD8 ($CD69^+CD8^+$) T cells. (i) Proliferating CD4 ($Ki67^+CD4^+$) and CD8 ($Ki67^+CD8^+$) T cells. **(j)** regulatory T ($CD25^+FoxP3^+CD4^+$) cells.

Supplementary Figure 6

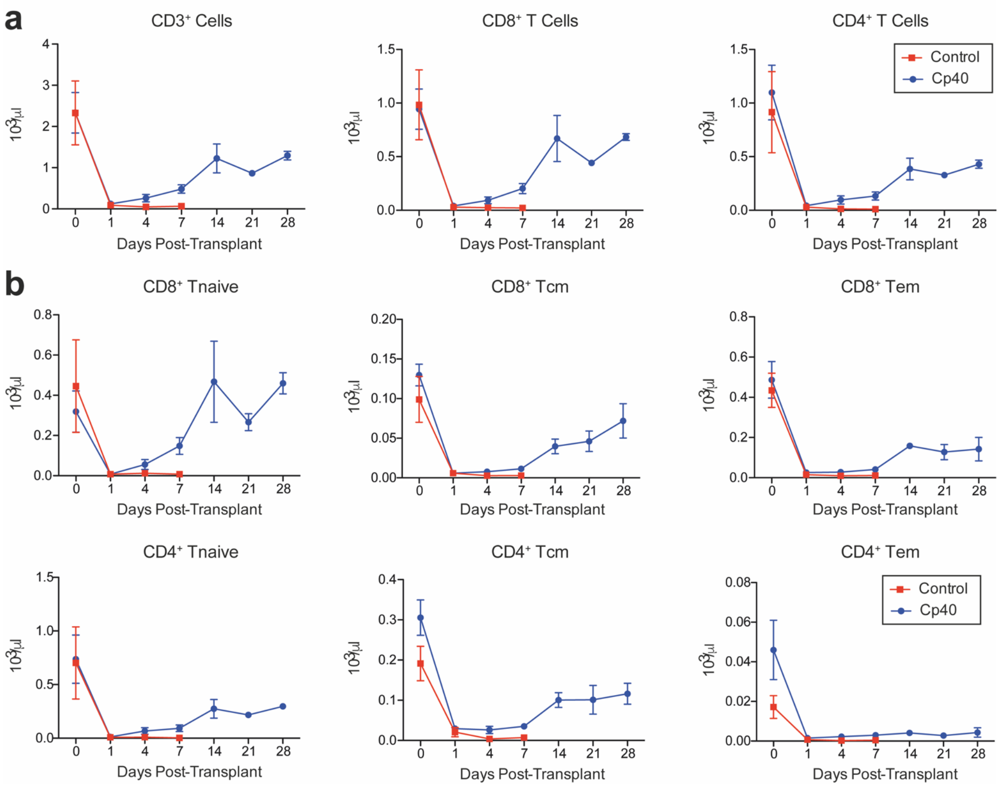

Supplementary Fig. 6: T cell depletion and repopulation. (a) Absolute cell counts of $CD3^+$ T cells and $CD4^+$ and $CD8^+$ T cell subsets in peripheral blood of the control group (red, n=5) and Cp40-treated group (blue, n=6). **(b)** Absolute cell counts of $CD4^+$ and $CD8^+$ T cell subsets of the control (red, n=5) and Cp40-treated (blue, n=6) groups defined by CD28 and CD95 (Tnaive= naïve T cells ($CD28^+$ $CD95^-$); Tcm= central memory T cells ($CD28^+$ $CD95^+$); Tem= effector memory T cells ($CD28^-$ $CD95^+$)). All data are presented as mean ± SD; N number is reflecting samples from biologically independent animals.

Supplementary Figure 7

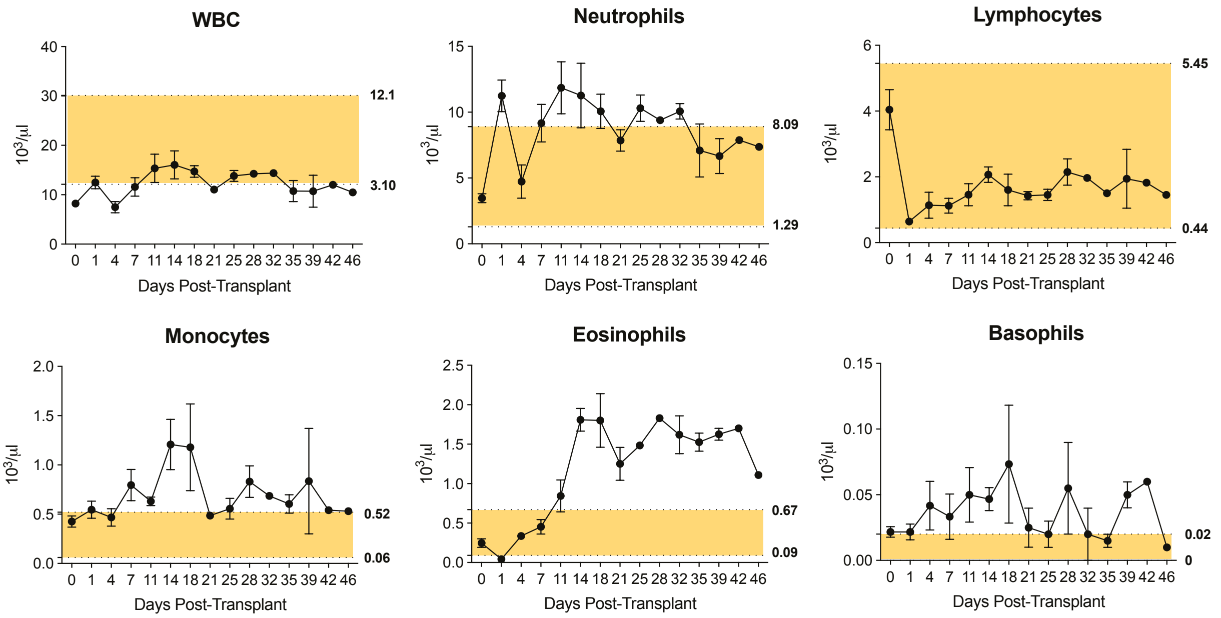

Supplementary Fig. 7: Post-transplant kinetics of immune cells of the Cp40-treated group (n=6). Hematological parameter values and normal ranges are indicated as colored area. All data are presented as mean ± SD.

Supplementary Figure 8

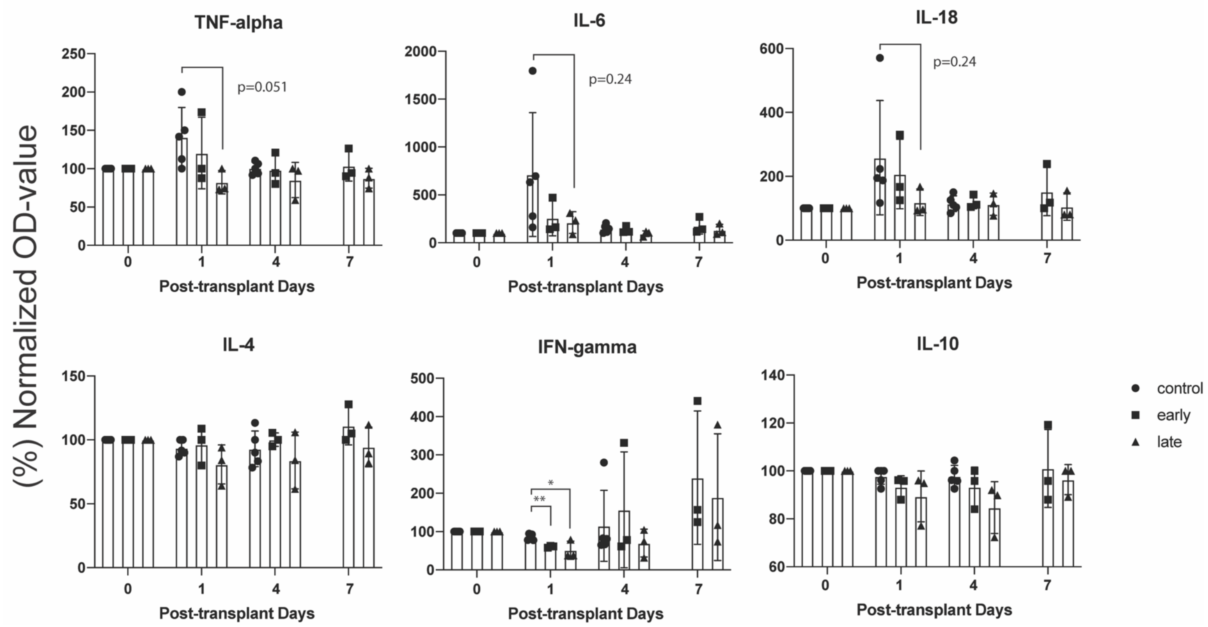

Supplementary Fig. 8: Multiplex cytokine analysis of serum from control and Cp40-treated animals. Serum level of TNF-α, IL-6, IL-18, IL-4, IFN-γ, and IL-10 were measured from control (n=3), ER (n=3), and LR (n=3) groups. Control animals showed significantly higher serum IFN-γ level compared to ER (p=0.0018) and LR (p=0.0193) groups at post-transplant day 1. All data are presented as mean ± SD; N number is reflecting samples from biologically independent animals. * indicates p<0.05; ** indicates p<0.01 using two-tailed parametric unpaired t test**.**

Supplementary Figure 9

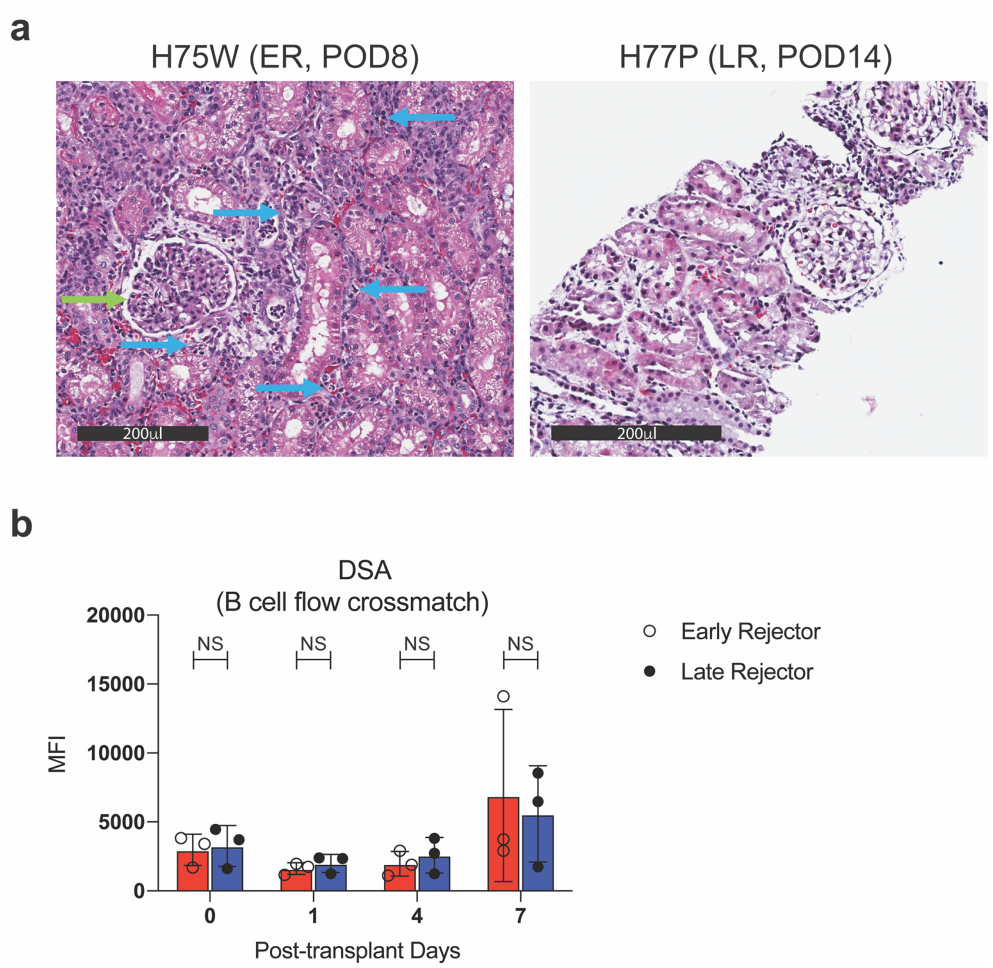

Supplementary Fig. 9: Early DSA kinetics and representative images for microcirculation inflammation in Cp40 treated animals (ER vs. LR). (a) H75W (ER, POD8) had glomerulitis (green arrow) and peritubular capillaritis (blue arrows) that was essentially absent in H77P (LR, POD14). **(b)** B cell flow crossmatch of the early post-transplant phase (day 0-7) of the ER (n=3) and LR (n=3) groups. Data are presented as mean ± SD. N number is reflecting samples from biologically independent animals; NS indicates no statistical significance using two-tailed parametric unpaired t test.

Supplementary Figure 10

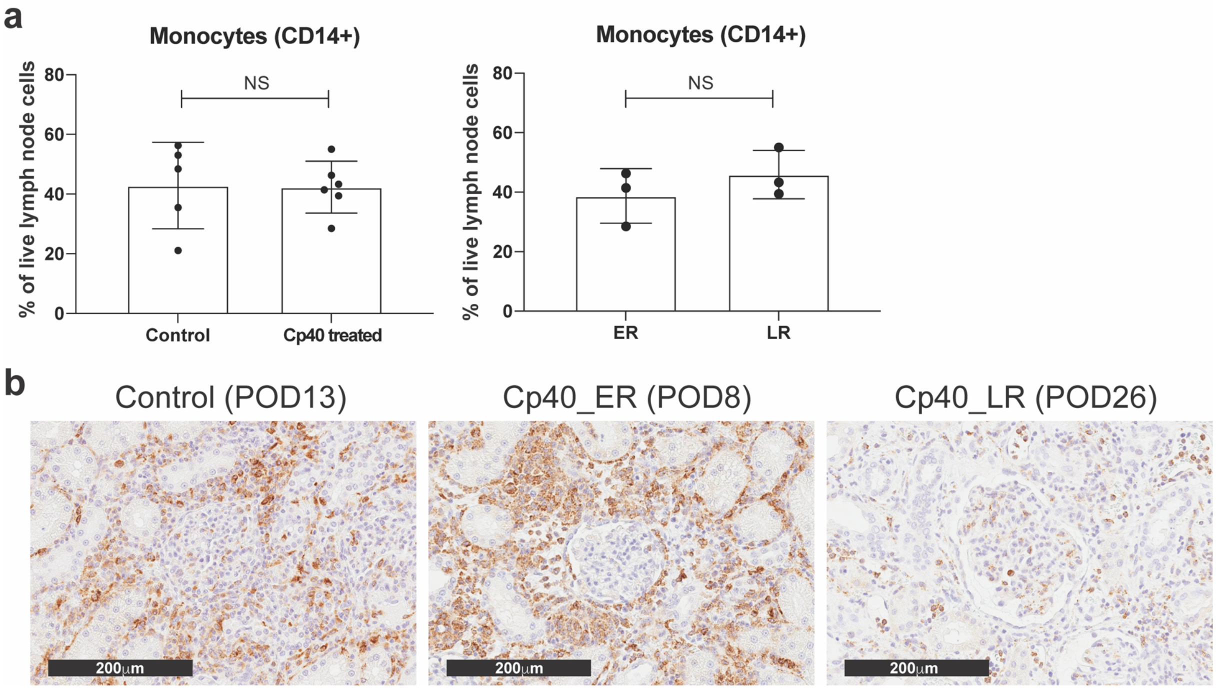

Supplementary Fig. 10: Lymph node monocytes and graft infiltrating macrophages. **(a)** Early post-transplant monocyte level in the lymph node. Monocytes from lymph nodes were analyzed in Control (n=5), ER (n=3) and LR (n=3) groups. Lymph node samples were collected either at necropsy (control and ER) or at POD28 (LR). Data are presented as mean ± SD. **(b)** Representative images for $CD68^{+}$ macrophage infiltration in the graft from control, ER, and LR. N number is reflecting samples from biologically independent animals; NS indicates no statistical significance using two-tailed parametric unpaired t test.

Supplementary Figure 11

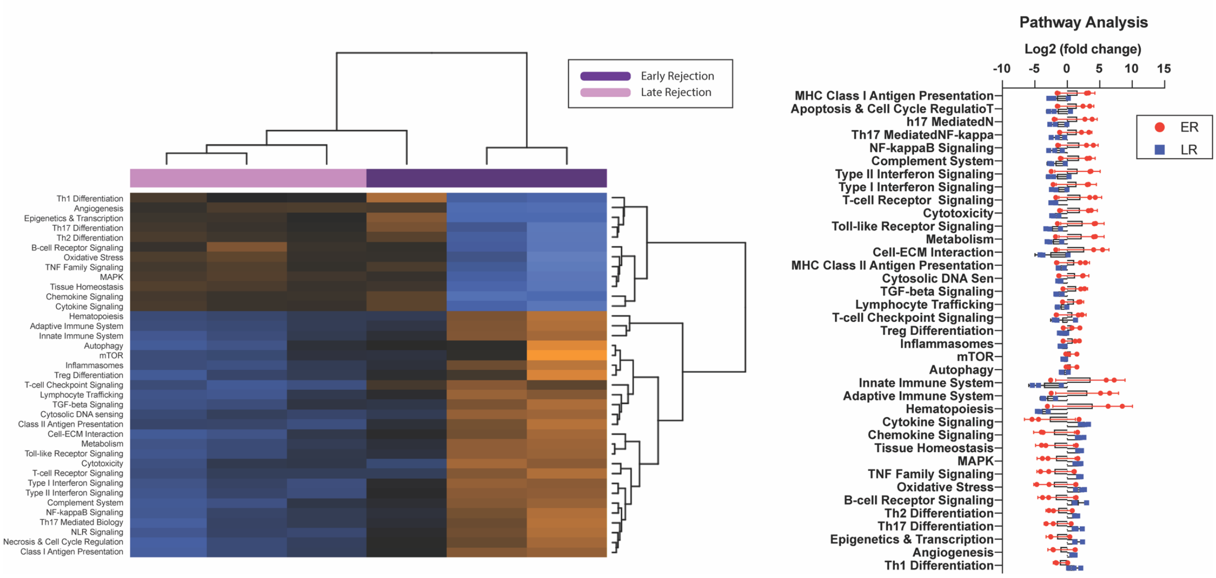

Supplementary Fig. 11. Hierarchical clustering of graft samples from Cp40-treated animal (n=6) using 771 genes and 37 pathways from human organ transplantation panel. The dendrogram and bar-graph are based on the Pathway Scoring module. The scores themselves are generated for each sample using a linear combination (weighted average) of the specific pathway's normalized gene expression values, weighing specific genes to capture the greatest possible variability in the data. This equates to the first Principal Component in gene expression for that pathway. These scores then undergo Z transformation to generate the dendrogram plot. The clustering is done using Pearson correlation. The algorithm for the Pathway Scoring module is based on previous study (34). Data are presented as mean ± SD.

Supplementary Table 1: MHC class I (MAMU-A and MAMU-B) and class II (MAMU-DRB, DQB, DPA, and DPB) haplotypes of all primates involved in this study. Primates marked in grey (H50R, H17M, H46G, H09W, and H58G) only served as donors for this study.

Animal ID	Mamu-A Haplotype 1	Mamu-A Haplotype 2	Mamu-B Haplotype 1	Mamu-B Haplotype 2	Mamu-DRB Haplotype 1	Mamu-DRB Haplotype 2	Mamu-DQA Haplotype 1	Mamu-DQA Haplotype 2	Mamu-DQB Haplotype 1	Mamu-DQB Haplotype 2	Mamu-DPA Haplotype 1	Mamu-DPA Haplotype 2	Mamu-DPB Haplotype 1	Mamu-DPB Haplotype 2	Mismatches
H50R (Donor)	A004	A001	B048	B047a	DR01a	DR04a	26g2	01g2	18g3	06_01	06g	02g1	01g1	15g1	
H49V	A002a	A006	B015a	B055	DR15a/b	DR03a	01g3	26g1	06g1	18g1	02g1	02g1	15g1	06_02	2-2-2-2-2-1-1
H17M (Donor)	A001	A004	B047a	B055	DR03a	DR03g	01g1	26g1	06g2	18g1	06g	02g2	01g1	07_01	
H764	A002a	A023	B015a	B001a	DR15a/b	DR03f	01g3	01g1	06g1	06g2	02g1	06g	15g1	01g1	2-2-2-2-2-1-2
H46G (Donor)	A004	A023	B001a	B043a	DR04a	DR06	01g2	23_01	06_01	06_01	02g1	02g1	15g1	15g1	
H60L	A002a	A105	B012a	B002	DR03f	DRb-unk	01g1	01g1	06g2	06g2	04g1	04g1	02_02	02_02	2-2-2-2-2-2-2
H09W (Donor)	A006	A004	B024a	B015a	DR10a	DR03a	26g3	26g1	15g	15g	02g1	02g2	08_01	07_01	
H42A	A001	A023	B055	B043a	DR03g	DR06	01g1	23_01	06g2	18_02	06g	02g1	01g1	15g1	2-2-2-2-2-1-2
H58G (Donor)	A004	A002a	B001a	B012a	DR03f	DR03f	01g1	01g1	06g2	06g2	07_01	07_01	19g	19g	
H57G	A023	A074	B043a	B001a	DR06	DR13a	23_01	24g2	18_02	15_02	02g1	04g2	15g1	02_01	2-1-2-2-2-2-2
H49G	A023	A004	B012b	B048	DR015c	DR01a	01g2	26g2	06_01	06_01	02g1	02g1	15g1	15g1	2-2-1-1-2-1-2
H75W	A002a	A008	B012a	B015a	DR03a	DR01a	26_01	26g2	18g3	18g1	02g1	02g3	15g	07_01	2-2-1-1-2-1-2
H72E	A002a	A001	B015	B055	DR15a/b	DR03g	01g2	01_02	06g1	06g2	02g1	06g	15g	01g1	2-2-2-2-2-2-2
H77P	A004	A019	B017a	B015c	DR10	DR03a	26g1	26_01	15g1	18g3	07_01	02g3	19_02	07_01	2-2-2-2-2-2-2
HADV	A025	A002a	B012b	B001a	DR03f	DR16	01_02	23_02	06g2	18g5	06g	08g	01g1	04_01	2-2-2-2-2-2-2
H87T	A019	A023	B015c	B043a	DR03a	DR06	26_01	23_01	18g3	18g4	02g3	02g1	07_01	15g	2-2-2-2-2-2-2

Supplementary Table 2: Flow cytometry antibody information

Parameter	Filter	Fluorochrome	Marker	Host	Manufacturer	Clone
Blue A	710/50	PerCP-CY5.5	CD4	Mouse	BD	L200
Blue A	710/50	PerCP-eFlour710	CD27	Mouse	invitrogen	O323
Blue A	710/50	PerCP-CY5.5	Ki-67	Mouse	BD	B56
Blue B	530/30	FITC	CD45RA	Mouse	BD	L48
Blue B	530/30	AlexaFluor 488	FoxP3	Mouse	BioLegend	259D
Blue B	530/30	FITC	IgD	Goat	SouthernBiotech	polyclonal
Red B	730/45	AlexaFluor 700	CCR7	Mouse	BD	150503
Red B	730/40	AlexaFluor 700	IgG	Mouse	BD	G18-145
Red B	730/45	AlexaFluor 700	CD20	Mouse	BD	2H7
Red C	670/30	APC	CD25	Mouse	invitrogen	CD25-3G10
Red C	780/60	APC-Cy7	CD8	Mouse	BD	RPA-T8
Red C	670/30	APC	CD127	Mouse	eBioscience	eBioRDR5
Red C	780/60	APC-Cy7	CD20	Mouse	BioLegend	2H7
Red C	670/30	APC	CD19	Mouse	Abcam	CB19
Red C	670/30	APC	PD-1	Mouse	eBioscience	eBioJ105
Violet E	525/50	V500	CD3	Mouse	BD	SP34-2
Violet E	525/50	BV510	CD14	Mouse	BD	M5E2
Violet F	450/50	eFlour450	CD95	Mouse	eBioscience	DX2
Violet F	450/50	PacBlue	IgM	Mouse	BioLegend	MHM-88
Violet F	450/50	PacBlue	CD8	Mouse	BD	RPA-T8
YGreen A	780/60	PE-Cy7	CD28	Mouse	eBioscience	CD28.2
YGreen A	780/60	PE-Cy7	ICOS	Hamster	BioLegend	C398.4A
YGreen D	586/15	PE	Ki-67	Mouse	BD	B56
YGreen D	586/15	PE	CD25	Mouse	Miltenyi Biotec	4E3
YGreen D	586/15	PE	CD38	Mouse	NHP Reagent Resource	OKT10
YGreen D	586/15	PE	CXCR5	Mouse	eBioscience	MU5UBEE

Supplementary Table 3: Banff Scoring of kidney allograft from Cp40 treated animals

Group	Case	POD	t	v	i	ti	i-IFTA	g	ci	ct	cg	mm	cv	ah	ptc	Diagnosis
ER	H75W	8	3	3	2	2	3	3	1	0	0	0	0	0	3	ACR, type 3 (focal arterial fibrinoid necrosis with mural inflammation); findings also suspicious for **AMR.**
ER	H49G	8	1	1	2	2	2	3	1	1	1b	3	3	0	2	ACR, type 2A; Findings also suspicious for **AMR.**
ER	HADV	12	3	3	2	2	0	3	1	1	1b	0	1	0	0	ACR, type 3 (focal arterial fibrinoid necrosis with mural inflammation & aneurysmal-type change). Findings are at least suspicious for **AMR.**
LR	H87T	28	1	0	0	0	0	0	1	1	0	0	0	0	2	"Borderline changes"; Findings also suspicious for **AMR.**
LR	H77P	14	0	0	0	0	0	0	0	0	0	0	0	0	0	No evidence of ACR. Mild tubular injury.
LR	H72E	14	0	0	0	0	0	0	0	0	0	0	0	0	0	No evidence of ACR. Mild tubular injury.

ACR = acute cellular rejection, AMR = antibody-mediated rejection.
