# Supplementary material for: C3 complement inhibition prevents antibody-mediated rejection and prolongs renal allograft survival in sensitized non-human primates
Source: Nat Commun. 2021 Sep 15;12:5456. doi: 10.1038/s41467-021-25745-7 (PMC8443599; doi:10.1038/s41467-021-25745-7)
Supplement: Supplementary file 3 — Reporting Summary [file 41467_2021_25745_MOESM3_ESM.pdf]

nature portfolio

Corresponding author(s): Jean Kwun and Stuart Knechtle

Last updated by author(s): 06-20-2021

Reporting Summary

Nature Portfolio wishes to improve the reproducibility of the work that we publish. This form provides structure for consistency and transparency in reporting. For further information on Nature Portfolio policies, see our Editorial Policies and the Editorial Policy Checklist.

Statistics

For all statistical analyses, confirm that the following items are present in the figure legend, table legend, main text, or Methods section.

n/a	Confirmed	
☐	☒	The exact sample size (*n*) for each experimental group/condition, given as a discrete number and unit of measurement
☐	☒	A statement on whether measurements were taken from distinct samples or whether the same sample was measured repeatedly
☐	☒	The statistical test(s) used AND whether they are one- or two-sided<br>*Only common tests should be described solely by name; describe more complex techniques in the Methods section.*
☒	☐	A description of all covariates tested
☒	☐	A description of any assumptions or corrections, such as tests of normality and adjustment for multiple comparisons
☐	☒	A full description of the statistical parameters including central tendency (e.g. means) or other basic estimates (e.g. regression coefficient) AND variation (e.g. standard deviation) or associated estimates of uncertainty (e.g. confidence intervals)
☐	☒	For null hypothesis testing, the test statistic (e.g. F, t, r) with confidence intervals, effect sizes, degrees of freedom and P value noted<br>*Give P values as exact values whenever suitable.*
☒	☐	For Bayesian analysis, information on the choice of priors and Markov chain Monte Carlo settings
☒	☐	For hierarchical and complex designs, identification of the appropriate level for tests and full reporting of outcomes
☒	☐	Estimates of effect sizes (e.g. Cohen's d, Pearson's r), indicating how they were calculated

Our web collection on statistics for biologists contains articles on many of the points above.

Software and code

Policy information about availability of computer code

Data collection	BD FACSDiva v8.0.2 (BD Biosciences), NanoString nCounter MAX platform
Data analysis	FlowJo v10 (BD Biosciences), FlowJo v9 (BD Biosciences), nSolver software platform 4.0, Grapad Prism 9.0

For manuscripts utilizing custom algorithms or software that are central to the research but not yet described in published literature, software must be made available to editors and reviewers. We strongly encourage code deposition in a community repository (e.g. GitHub). See the Nature Portfolio guidelines for submitting code & software for further information.

Data

Policy information about availability of data

All manuscripts must include a data availability statement. This statement should provide the following information, where applicable:

- Accession codes, unique identifiers, or web links for publicly available datasets
- A description of any restrictions on data availability
- For clinical datasets or third party data, please ensure that the statement adheres to our policy

The primary data that support the findings of this study are available from the corresponding author upon reasonable request. Supplementary information accompanies this paper and the nanostring data have been deposited in NCBI's Gene Expression Omnibus (GEO) and are accessible through GEO Series accession number GSE178843.

Field-specific reporting

Please select the one below that is the best fit for your research. If you are not sure, read the appropriate sections before making your selection.

☒ Life sciences ☐ Behavioural & social sciences ☐ Ecological, evolutionary & environmental sciences

For a reference copy of the document with all sections, see nature.com/documents/nr-reporting-summary-flat.pdf

Life sciences study design

All studies must disclose on these points even when the disclosure is negative.

Sample size	We have undertaken a rigorous analysis of group size for these transplants, so that that our experiments are adequately powered to make be statistically and clinically relevant predictions. Group sizes were determined based on both a time-to-onset variable, measuring the first sign of rejection (or desensitization) in untreated controls versus experimental cohorts as well as on the expected rate of rejection (or degree of desensitization ~50%) in these two cohorts. Based on our preliminary studies, the most accurate estimate of time-to-onset rejection in control cohorts (without desensitization) is between 1-9 days. these estimates, adequate group size calculations yield group number of 3-5 animals per group. For budgeting purposes, we have estimated 5-6 animals per group for each treatment regimen.
Data exclusions	In figure 2A (multiplex cytokine assay), data were excluded if the %CV of the duplicate is >20%. No other data were excluded.
Replication	The outcome of in vivo experiments (NHP kidney transplantation) with anti-C3 treatment was subdivided into two phenotypes (Early Rejector vs. Late Rejector). We suggested that the high level of preformed IgM DSA prior to kidney transplantation make animals to resistant to anti-C3 approach.
Randomization	Samples were allocated into experimental groups randomly.
Blinding	Blinding was not possible - Investigators participated in surgery (renal transplantation), post-op care, and post-op treatment. Tissue samples were graded by the transplant pathologist blindly.

Reporting for specific materials, systems and methods

We require information from authors about some types of materials, experimental systems and methods used in many studies. Here, indicate whether each material, system or method listed is relevant to your study. If you are not sure if a list item applies to your research, read the appropriate section before selecting a response.

Materials & experimental systems

n/a	Involved in the study	
☐	☒	Antibodies
☒	☐	Eukaryotic cell lines
☒	☐	Palaeontology and archaeology
☐	☒	Animals and other organisms
☒	☐	Human research participants
☒	☐	Clinical data
☒	☐	Dual use research of concern

Methods

n/a	Involved in the study	
☒	☐	ChIP-seq
☐	☒	Flow cytometry
☒	☐	MRI-based neuroimaging

Antibodies

Antibodies used

Fluorochrome -- Marker -- Host -- Manufacturer -- Clone
PerCP-CY5.5 -- CD4 -- Mouse -- BD --L200
PerCP-eFlour710 --CD27 --Mouse --invitrogen --O323
PerCP-CY5.5 --Ki-67 --Mouse --BD --B56
FITC --CD45RA --Mouse --BD --L48
AlexaFluor 488 --FoxP3 --Mouse --BioLegend --259D
FITC --IgD --Goat --SouthernBiotech --polyclonal
AlexaFluor 700 --CCR7 --Mouse --BD --150503
AlexaFluor 700 --IgG --Mouse --BD --G18-145
AlexaFluor 700 --CD20 --Mouse --BD --2H7
APC --CD25 --Mouse --invitrogen --CD25-3G10
APC-Cy7 --CD8 --Mouse --BD --RPA-T8
APC --CD127 --Mouse --eBioscience --eBioRDR5
APC-Cy7 --CD20 --Mouse --BioLegend --2H7
APC --CD19 --Mouse --Abcam --CB19
APC --PD-1 --Mouse --eBioscience --eBioJ105
V500 --CD3 --Mouse --BD --SP34-2

BV510 --CD14 --Mouse --BD --M5E2
eFlour450 --CD95 --Mouse --eBioscience --DX2
PacBlue --IgM --Mouse --BioLegend --MHM-88
PacBlue --CD8 --Mouse --BD --RPA-T8
PE-Cy7 --CD28 --Mouse --eBioscience --CD28.2
PE-Cy7 --ICOS --Hamster --BioLegend --C398.4A
PE --Ki-67 --Mouse --BD --B56
PE --CD25 --Mouse --Miltenyi Biotec --4E3
PE --CD38 --Mouse --NHP Reagent Resource --OKT10
PE --CXCR5 --Mouse --eBioscience --MU5UBEE

Validation

All antibodies were commercially available and validated by manufacturers. All antibodies were titrated using rhesus PBMC or splenocytes prior to establishment of staining protocol.

Animals and other organisms

Policy information about studies involving animals; ARRIVE guidelines recommended for reporting animal research

Laboratory animals

Species -- Strain -- Sex -- Age
Nonhuman Primate -- Rhesus Macaque -- Male -- 3~6 years old

Wild animals

N/A

Field-collected samples

N/A

Ethics oversight

All animal care and procedures were conducted in accordance with the National Institutes of Health (NIH) guidelines and were approved by the Institutional Animal Care and Use Committee.

Note that full information on the approval of the study protocol must also be provided in the manuscript.

Flow Cytometry

Plots

Confirm that:

☒ The axis labels state the marker and fluorochrome used (e.g. CD4-FITC).

☒ The axis scales are clearly visible. Include numbers along axes only for bottom left plot of group (a 'group' is an analysis of identical markers).

☒ All plots are contour plots with outliers or pseudocolor plots.

☒ A numerical value for number of cells or percentage (with statistics) is provided.

Methodology

Sample preparation

Blood was collected in EDTA tubes for PBMCs and plasma, clot tube for serum collection via femoral vein. PBMCs were obtained by density-gradient centrifugation using 90% separation media from Sigma. Samples were resuspended in in PBS + 2% fetal calf serum to create single cell suspensions. Peripheral lymph node (LN) biopsies were ground over 70 micrometer cell strainer to obtain LN-derived cells in PBS + 2% fetal calf serum. For flow crossmatch, recipient serum samples were incubated with donor peripheral blood mononuclear cells (PBMCs) and then resuspneded in PBS + 2% fetal calf serum.

Instrument

BD LSRFortessa (BD Biosciences)

Software

BD FACSDiva (BD Biosciences) was used for data collection. FlowJo v9 or 10 (BD Biosciences) were used for manual gating.

Cell population abundance

No cells were sorted during this study.

Gating strategy

Figure 1D, Figure 4B and 6A (B cell flow crossmatch)
Forward scatter area vs. side scatter area --> forward scatter area vs. forward scatter height (singlet)--> forward scatter area vs. live/dead cells (Aqua)--> Forward scatter area vs. side scatter area (lymphocytes)--> CD3 vs. CD20 --> IgG histogram, IgM histogram

Figure 2A and 2B, 5C, 5D, and 5E (B cell and T cell phenotypes)
Forward scatter area vs. side scatter area --> forward scatter area vs. forward scatter height (singlet)--> forward scatter area vs. live/dead cells (Aqua)--> Forward scatter area vs. side scatter area (lymphocytes)--> CD3 vs. CD20 --> CD20 vs. Ki67, CD4 vs. Ki67, CD8 vs. Ki67, CD4/CD8 --> CD28 vs. CD95, FoxP3 vs. CD25.

☒ Tick this box to confirm that a figure exemplifying the gating strategy is provided in the Supplementary Information.
